# Role of clinical parameters and early noncontrast magnetic resonance imaging scan of brain in prediction of final neurologic outcome of hanging victims

**DOI:** 10.4103/0974-2700.66548

**Published:** 2010

**Authors:** Srijan Mazumdar, Priyam Mukherjee, Soumik Goswami, Jotideb Mukhopadhyay

**Affiliations:** Department of Medicine, Institute of Post Graduate Medical Education and Research & SSKM Hospital, Kolkata, West Bengal, India

Sir,

Hanging is a common mode of suicide worldwide. However, almost 80% survive, often with permanent neurosequelae due to parenchymal brain damage. We tried to correlate clinical parameters and results of noncontrast magnetic resonance imaging (MRI) of brain with development of neurologic deficits in patient presenting post hanging. Neurodeficits studied were the delayed development of seizure, hemiparesis or movement disorders within a period of 6 months post discharge from the hospital.

We included 19 cases in our study (out of which 10 were males and 9 were females). Data regarding Glasgow Coma Scale (GCS), systolic and diastolic blood pressure, pulse rate, respiratory rate, plantar and pupillary reflex were collected retrospectively from patient charts on all the 19 patients. Noncontrast MRI of brain was done within 48 hours of incidence of hanging, irrespective of duration of hanging on all the patients. We excluded patients with a past history of seizure, stroke or movement disorder. Out of the 19 patients, 7 (36.8%) developed delayed neurosequelae. Two patients (28.57%) developed hemiparesis, four patients (57.14%) developed seizure disorder, while one patient (14.28%) developed hemichorea. The interesting part about these patients was that all of them had a normal noncontrast MRI of brain done within 48 hours of admission.

GCS value below 10 at admission was associated with adverse neurological outcome (*P* = 0.035). Abnormal plantar reflex, defined as unilateral or bilateral equivocal or extensor response, strongly correlated with neurosequelae development (*P* = 0.015). Pulse rate (*P* = 0.516), respiratory rate *P* = 0.340), systolic blood pressure *P* = 0.336), diastolic blood pressure (*P* = 0.912), pupil size (*P* = 0.054) and pupillary reaction to light had insignificant association with future neurosequelae as was the case with non-contrast MRI.

In hanging, the brain parenchymal damage results from either venous congestion in brain leading to hypoxic neuronal injury or hemorrhage from rupture of vessels. Hypoxia can also occur from bilateral carotid compression at neck with severe constriction pressure. This ultimately results in damage to the basal ganglia, thalamus and cortex of the brain, with development of motor neurosequelae. It is clear from our study that in case of hanging, clinical parameters at presentation, in particular GCS score below 10 and upgoing or nonresponsive plantar reflex, have significant correlation in predicting future neurosequelae.[1–3] Pupil size may also be useful for the purpose. MRI scan of brain without contrast had no value in our patients but cannot be excluded, not to forget the challenges of its limited availability, high cost and possible aggravation of neck injury during transportation of the patient to MRI unit.[4,5] Contrast MRI or Diffusion Weighted Images(DWI) images of brain may theoretically pick up the brain tissue injury early as seen in hypoxic brain damage in neonates. Magnetic Resonance Angiography(MRA)of neck vessels and/or brain was not done due to logistic problem and again theoretically it is better in identifying early lesion. Further studies are needed to address these issues.
